# Hepatic Hemodynamics and Fetal Growth: A Relationship of Interest for Further Research

**DOI:** 10.1371/journal.pone.0115594

**Published:** 2014-12-23

**Authors:** Sharona Vonck, Anneleen Simone Staelens, Tinne Mesens, Kathleen Tomsin, Wilfried Gyselaers

**Affiliations:** 1 Faculty of Medicine and Life Sciences, Hasselt University, Hasselt, Belgium; 2 Department of Obstetrics & Gynaecology, Ziekenhuis Oost-Limburg, Genk, Belgium; 3 Department Physiology, Hasselt University, Hasselt, Belgium; National Taiwan University Hospital, Taiwan

## Abstract

**Background:**

It is well known that hepatic hemodynamics is an important physiologic mechanism in the regulation of cardiac output (CO). It has been reported that maternal cardiac output relates to neonatal weight at birth.

**Aims:**

In this study, we assessed the correlation between maternal hepatic vein Doppler flow parameters, cardiac output and neonatal birth weight.

**Methods:**

Healthy women with uncomplicated second or third trimester pregnancy attending the outpatient antenatal clinic of Ziekenhuis Oost-Limburg in Genk (Belgium), had a standardized combined electrocardiogram-Doppler ultrasound with Impedance Cardiography, for measurement of Hepatic Vein Impedance Index (HVI  =  [maximum velocity – minimum velocity]/maximum velocity), venous pulse transit time (VPTT  =  time interval between corresponding ECG and Doppler wave characteristics) and cardiac output (heart rate x stroke volume). After delivery, a population-specific birth weight chart, established from a cohort of 27000 neonates born in the index hospital, was used to define customized birth weight percentiles (BW%). Correlations between HVI, VPTT, CO and BW% were calculated using Spearman's ρ, linear regression analysis and R^2^ goodness of fit in SPSS 22.0.

**Results:**

A total of 73 women were included. There was a negative correlation between HVI and VPTT (ρ = −0.719, p<0.001). Both HVI and VPTT correlated with CO (ρ = −0.403, p<0.001 and ρ = 0.332, p<0.004 resp.) and with BW% (ρ = −0.341, p<0.003 and ρ = 0.296, p<0.011 resp.)

**Conclusion:**

Our data illustrate that the known contribution of hepatic hemodynamics in the regulation of cardiac output is also true for women with uncomplicated pregnancies. Our study is the first to illustrate a potential link between maternal hepatic hemodynamics and neonatal birth weight. Whether this link is purely associative or whether hepatic vascular physiology has a direct impact on fetal growth is to be evaluated in more extensive clinical and experimental research.

## Introduction

One of the main functions of the venous system is the regulation of cardiac output. The cardiovascular circuit is a closed circulatory loop, implicating that in steady state conditions the venous flow back to the heart (i.e. preload) is equal to the arterial flow towards the organs (i.e. cardiac output). Due to the Frank-Starling mechanism, an increase in venous return will automatically lead to an increase in cardiac output and vice versa. Next to this, the venous compartment is also a capacitance reservoir: storage of non-circulating reserve blood occurs in the splanchnic bed and liver, from where this can be mobilised into the circulation whenever necessary. This mobilization occurs via the portal vein and the liver. As such, the hepatic circulation is actively involved in the regulation of cardiac output, both in a direct and indirect way [1].

Changes of maternal cardiac output start already in early gestation [2]–[4]. The physiologic increase of cardiac output during pregnancy is important for a normal course of pregnancy. Several studies have highlighted the important interaction between maternal cardiovascular maladaptation (i.e. low cardiac output) and intra-uterine growth restriction (IUGR) with or without maternal hypertension or organ-dysfunction [5]–[7]. Bamfo et al. suggests that cardiac output is reduced in the IUGR population due to a reduction in stroke volume, which is the consequence of a reduction in preload [8].

An integrated assessment of the cardiovascular loop can be done non-invasively by the use of impedance cardiography (ICG) and combined electrocardiogram – Doppler (ECG-D) sonography [9]–[12]. ICG is a safe and reproducible technique for an estimation of cardiac output and other hemodynamic parameters [13]. Changes in maternal arterial and central venous function can be evaluated reliably by ECG-Doppler assessments [14]–[16].

The aim of the study was to investigate whether a relation exists between maternal ICG and/or ECG-D parameters and neonatal birth weight percentiles. As hepatic hemodynamics has a physiological role in the regulation of CO and CO is known to correlate with BW%, we hypothesize that HVI correlates with BW%.

## Methods

### Ethics Statement

The study protocol was approved by the local Ethics Committee of Hasselt University and Hospital Oost-Limburg (CME ZOL reference 08/049 and 09/050). Written informed consent was obtained.

### Participants

**Figure 1:**
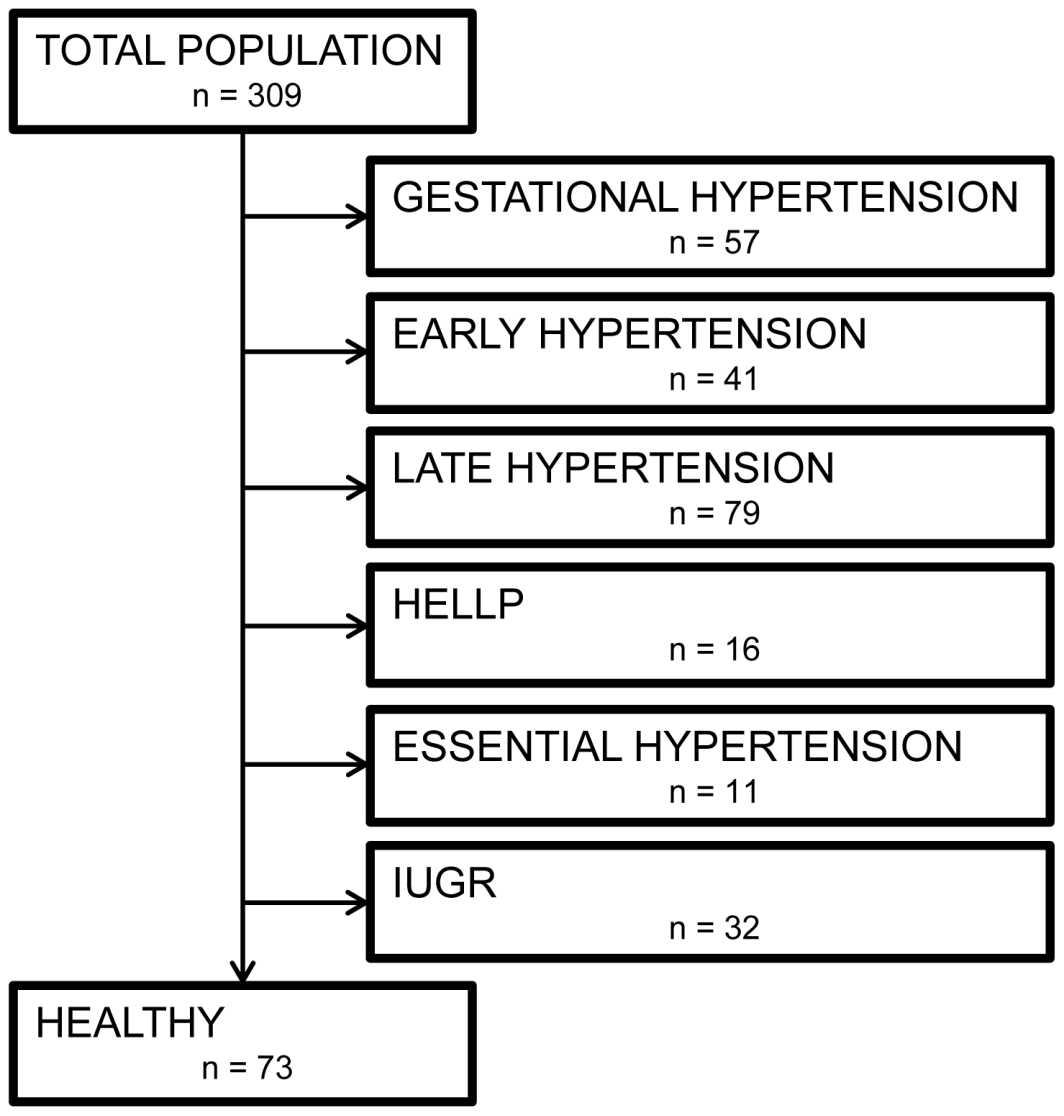
Structured record of the included and excluded participants.

Pregnant women in their second or third pregnancy trimester with or without gestational complications, attending the outpatient antenatal clinic, were invited to participate in this observational study. After written informed consent, a non-invasive cardiovascular assessment was performed according to a standardised protocol as reported elsewhere [11] using non-invasive impedance cardiography (ICG) and combined electrocardiogram-Doppler (ECG-D) ultrasound. Demographic details were recorded: maternal age (years), parity, pregestational BMI and gestational age at assessment. After delivery, outcome of pregnancy was evaluated, gestational age at birth and neonatal birth weight were also recorded. Only data from uncomplicated pregnancies with normal neonatal outcome were included in this study. Exclusion criteria were pre-existing maternal disease or medication use, diagnosis of preeclampsia, Haemolysis Elevated Liver enzymes and Low Platelets (HELLP), essential hypertension, gestational hypertension, multiplets and intra-uterine growth retardation (IUGR) (Fig. 1).

### Birth Weight percentiles

Customized birth weight charts were established from a cohort of 27000 neonates, born as singletons without congenital anomalies in Ziekenhuis Oost-Limburg between 2001 and 2013. Charts were categorized into 4 groups: primiparous & baby girl, primiparous & baby boy, multiparous & baby girl, multiparous & baby boy. Birth weights were classified per week of gestation, and birth weight percentiles (BW%) were calculated with an interval of 2.5% between P2.5 and P97.5. According to these population-specific data, the weight at birth of each neonate in the study was expressed as a customized birth weight percentile.

### Impedance Cardiography

ICG examinations were performed in standing position using the Non-Invasive Continuous Cardiac Output Monitor (NICCOMO, SonoSite, Medis Medizinische Messtechnik GmbH, Ilmenau, Germany), according to a standard protocol as described [17]. Technological principles, benefits, limitations and figures on reproducibility have been reported elsewhere [13], [18]. Maternal cardiac output (mL/min) was calculated from the product of measured values of heart rate (beats/min) and stroke volume (mL).

### Venous ECG-Doppler Ultrasound

The ECG-Doppler assessment was performed by four ultra-sonographers (SV, AS, TM and KT) with known intra- and interobserver correlation [16] according to the study protocol as reported [15] in supine position during interrupted breathing, using a 3.5 MHz transabdominal probe (Aplio Mx, Toshiba Medical Systems nv., Sint-Stevens-Woluwe, Belgium). The hepatic veins were evaluated at the cranio-caudal portion of the liver. Maximum en minimum velocities (V_max_, V_min_) were measured, and a hepatic venous impedance index (HVI) was calculated as [V_max_-V_min_/V_max_]. Venous pulse transit times (VPTT) were measured as the time interval between the P-wave of the ECG and the corresponding A-wave of the venous Doppler wave, corrected for heart rate [PA/RR] [14], [15].

### Statistics

Statistical analyses were done using SPSS 22.0. Shapiro Wilk was performed to assess normal distribution of VI, VPTT, CO and BW%. Data were recorded as median + interquartile range. Rank-based Spearman's ρ correlation coefficient was calculated to assess the relation between HVI, CO, VPTT and BW%. For each relation, linear regression analysis was performed with calculation of R^2^ goodness of fit and p-value, which was considered significant at nominal level α <0.05.

## Results

From a total of 309 women, 236 pregnant women were excluded due to maternal, gestational or fetal problems [11], leaving 73 inclusions with a normal course of pregnancy and neonatal outcome (Fig. 1). Data on participant demographics, neonatal outcome + BW% and maternal cardiovascular characteristics are shown in Table 1. Only CO and VPTT showed normal distribution.

**Table 1:** Demographic data, neonatal outcome and maternal cardiovascular characteristics in healthy pregnant women.

	Uncomplicated Pregnancies
	(n = 73)
Demographic characteristics	
Maternal age, years	29.74±5.77
Gestational age at inclusion, weeks	35w4d±5w4d
Pre-pregnancy BMI, kg/m^2^	23.38±6.06
Nulliparity, %	47.94
Neonatal outcome	
Birth weight, g	3325±725
Birth weight, percentile	50±52.5
Gestational age at delivery, weeks	39w1d±2w5d
Cardiovascular characteristics	
HVI	0.24±0.26
CO	7.8±2.2
VPTT	0.39±0.17

Data are presented as median ± interquartile range.

HVI: hepatic vein index, CO: cardiac output, VPTT: venous pulse transit time.

**Figure 2:**
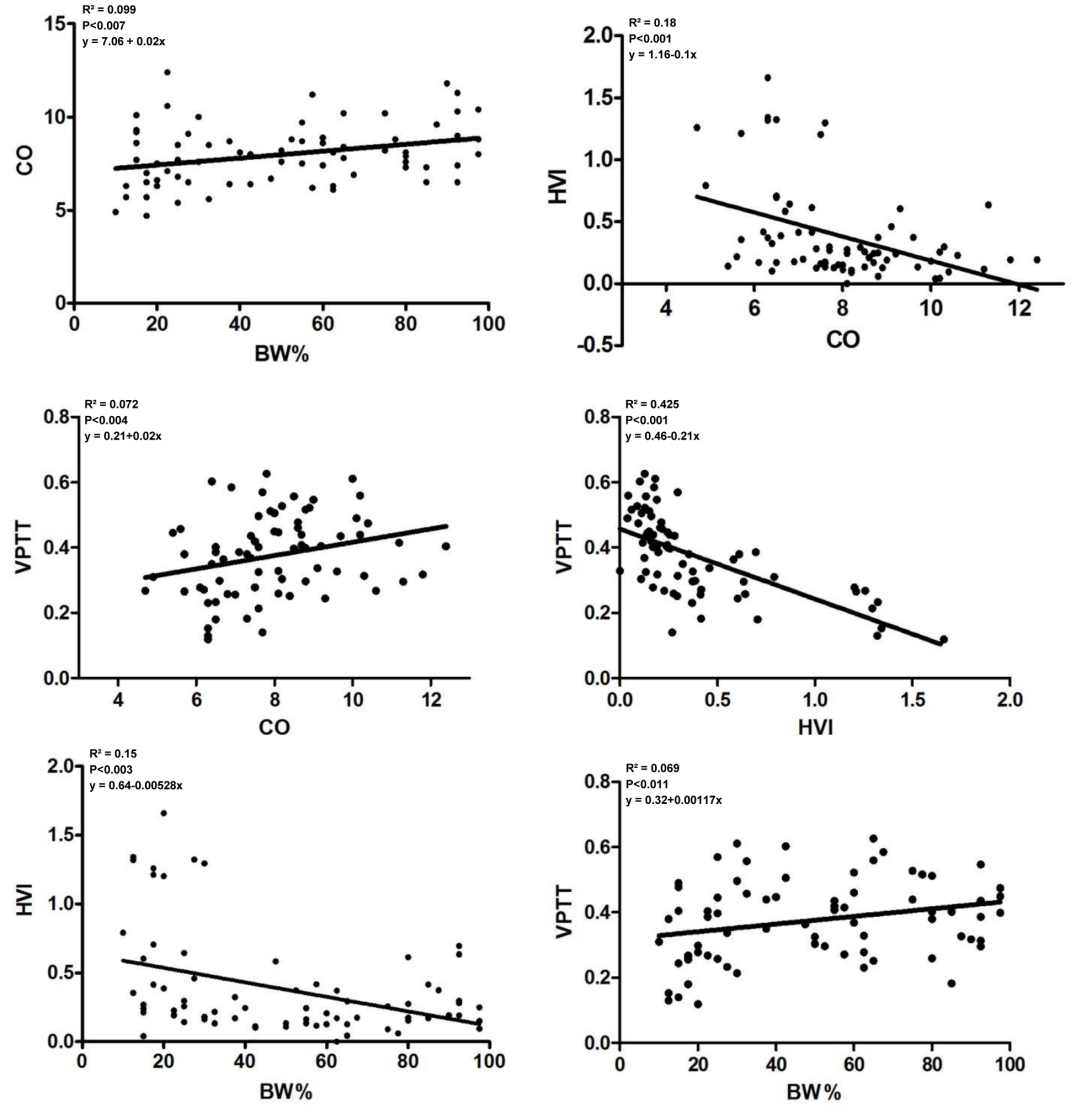
Scatterplots of the relations between maternal cardiac output (CO), hepatic vein impedance index (HVI), hepatic vein pulse transit time (VPTT) and customised birth weight percentile (BW%).

Correlations between HVI, VPTT, CO and BW% are shown in Fig. 2. All correlations were significant. There was a negative correlation between HVI and VPTT (ρ = −0.719, p<0.001). Both HVI and VPTT correlated with CO (ρ = −0.403, p<0.001 and ρ = 0.332, p<0.004 resp.) and with BW% (ρ = −0.341, p<0.003 and ρ = 0.296, p<0.011 resp.). Linear equations, values of R^2^ and p values are presented in Fig. 2.

## Discussion

This study aimed to investigate the relation between maternal cardiac output, maternal hepatic venous function and birth weight percentiles during healthy pregnancy. We observed that HVI and VPTT (hepatic venous parameters), both measured by ECG-Doppler, correlate with maternal CO as measured by ICG, which in turn correlates with birth weight percentiles. Moreover, the hepatic venous parameters also show a significant correlation with the birth weight percentiles.

This study's strengths are the use of rigid protocols for ICG and ECG-D assessment with reliable measurements and known inter- and intra-observer correlations, as well as customized population-specific birth weight charts. As all included women had antenatal care and delivery in the same hospital, all data on gestational, maternal and neonatal outcome are complete and directly available from the hospital records. The use of ICG for measuring maternal cardiac output during pregnancy may be subject of criticism [19], [20], however this point of discussion has been dealt with in a recent review from our research team [13]. The current analysis does however not permit that hepatic circulation per se impacts on birth weight since there is an interdependency between CO and hepatic circulation. A different analysis is necessary to assess the contribution of CO and VPTT/HVI to variations in BW%.

An influence of the maternal CO on birth weight is already observed in other studies, supporting a physiological role of maternal cardiac output on fetal growth [8], [21]–[23]. Already in the first trimester of pregnancy the relation between cardiac output and birth weights could be demonstrated [5], [12]. The maternal cardiac output starts to increase very early in pregnancy and pregnancies which fail to achieve this increase often end up with growth restricted babies [24].

The venous compartment cooperates with the heart as one functional unit to regulate venous return and cardiac output [25], [26]. This venous activity is far more important than the arterial resistance in this process [25]: experiments showed that changes of venous resistance affect the cardiac output eight times more than changes in arterial resistance [27]. A small change in vein diameter, compliance and intraluminal pressure has a tremendous impact on its blood content and flow, which can easily influence cardiac output [26]. Next to this, the venous compartment is a capacitance reservoir which contains approximately two thirds of the total blood volume, one third of this residing in the splanchnic bed [28]. The liver is considered the most important buffer system for acute intravascular volume changes: a sudden increase of blood volume is sequestered in the hepatic venous bed [29] whereas this stored volume can easily be mobilized into the systemic circulation in case of sudden blood loss [30]. As such, hepatic hemodynamics is an important physiologic system in the control and regulation of cardiac output. Our data are in line with this. On the one hand, we observed a positive correlation between venous pulse transit time (VPTT) of the liver and cardiac output. Venous pulse transit time is considered a Doppler measure for venous tone or wall stiffness. A low VPTT value suggests a fast retrograde conduct into the central circulation of the venous A-wave, which is caused by the contraction of the right atrium. This suggest a more rigid state of the venous vascular wall [15]. In chronic conditions, this low compliance will hamper intraluminal storage of blood and venous drainage from the organs [31], and consequently venous return and cardiac output will be low [25]. On the other hand, we observed that the hepatic vein impedance index (HVI) is negatively correlated with the cardiac output. This index is the Doppler equivalent of Arterial Resistivity Index and is calculated from the maximum and minimum velocities of the venous pulse wave [9]. A large value for HVI indicates a strong intravenous rebound of atrial contraction, which counteracts venous drainage from the organs [31]. In healthy pregnancies, this index becomes gradually lower due to adaptational changes [32]. Our findings illustrate that a low venous impedance index is correlated with a high cardiac output and vice versa. Both correlations suggest that the physiologic function of the hepatic venous bed in the regulation of cardiac output, reported for non-pregnant individuals, is also present during pregnancy. The importance of hepatic hemodynamics in normal or abnormal changes of maternal cardiac output is to be examined in further clinical and experimental research.

A new and interesting observation from our study is the correlation between hepatic venous Doppler indices and neonatal birth weight percentile (Fig. 2). From the arguments outlined above, this correlation in fact seems logical as hepatic hemodynamics is involved in control of cardiac output, and maternal cardiac output is known to correlate with birth weight percentiles. A significant correlation does not necessarily mean that a causal relation exists between hepatic venous flow and fetal growth, however our observation invites to further explore the role of hepatic hemodynamics in the process of maternal cardiovascular adaptation and in the pathophysiology of gestational complications such as fetal growth restriction, gestational hypertension and/or liver diseases.

From the data presented in this paper, we conclude that in healthy women with uncomplicated pregnancies a correlation exists between hepatic vein Doppler parameters, maternal cardiac output and fetal birth weight percentiles. The role of hepatic hemodynamics in the regulation of cardiac output of non-pregnant individuals, and the relevance of maternal cardiac output towards fetal growth are well known. However our data show for the first time a potential link between maternal hepatic hemodynamics and neonatal weight at birth. Whether this link is purely associative or whether hepatic vascular physiology has a direct impact on fetal growth is to be evaluated in more extensive clinical and experimental research.
